# Evaluation of hospital inpatient complications: a planning approach

**DOI:** 10.1186/1472-6963-10-200

**Published:** 2010-07-09

**Authors:** Ronald J Lagoe, Gert P Westert

**Affiliations:** 1 Hospital Executive Council Syracuse, New York, USA; 2 National Institute for Public Health and the Environment Bilthoven, Netherlands

## Abstract

**Background:**

Hospital inpatient complications are one of a number of adverse health care outcomes. Reducing complications has been identified as an approach to improving care and saving resources as part of the health care reform efforts in the United States.

An objective of this study was to describe the Potentially Preventable Complications software developed as a tool for evaluating hospital inpatient outcomes. Additional objectives included demonstration of the use of this software to evaluate the connection between health care outcomes and expenses in United States administrative data at the state and local levels and the use of the software to plan and implement interventions to reduce hospital complications in one U.S. metropolitan area.

**Methods:**

The study described the Potentially Preventable Complications software as a tool for evaluating these inpatient hospital outcomes. Through administrative hospital charge data from California and Maryland and through cost data from three hospitals in Syracuse, New York, expenses for patients with and without complications were compared. These comparisons were based on patients in the same All Patients Refined Diagnosis Related Groups and severity of illness categories. This analysis included tests of statistical significance.

In addition, the study included a planning process for use of the Potentially Preventable Complications software in three Syracuse hospitals to plan and implement reductions in hospital inpatient complications. The use of the PPC software in cost comparisons and reduction of complications included tests of statistical significance.

**Results:**

The study demonstrated that Potentially Preventable Complications were associated with significantly increased cost in administrative data from the United States in California and Maryland and in actual cost data from the hospitals of Syracuse, New York. The implementation of interventions in the Syracuse hospitals was associated with the reduction of complications for urinary tract infection, decubitus ulcer, and pulmonary embolism.

**Conclusions:**

The study demonstrated that the Potentially Preventable Complications software could be used to evaluate hospital outcomes and related costs at the aggregate and diagnosis specific levels. It also indicated that the system could be used to plan and implement interventions to improve outcomes on an individual or multihospital basis.

## Background

Interest in the improvement of quality and outcomes in health care is increasing in both Europe and the United States. Initially, this development was driven mainly by attention to improving care at the patient level [1]. More recently, however, a linkage between improving outcomes and saving health care costs has developed.

An important study evaluated the direct medical costs of a wide range of adverse events in hospitals in the Netherlands. This study included unplanned admissions; unplanned readmissions; hospital acquired complications including infections, neurological complications, and other diagnoses; unexpected mortality, and other issues. A study of a wide range of adverse medical events in hospitals in the Netherlands suggested that 30,000 inpatient admissions included a preventable adverse event. On the basis of data collected in 2004, it estimated that the annual costs of preventable adverse events in Dutch hospitals was 161 million euros [2]. Other European studies have evaluated potentially preventable mortality in Dutch hospitals [3], hospital acquired urinary tract infections [4], nosocomial pneumonia [5,6], hospital acquired infections [7], pressure ulcers [8], and adverse events in British Hospitals [9]. The Hoonhout and Zegers studies were carried out through the Netherlands adverse events research and used the same databases.

Studies have also evaluated the financial impact of adverse health care outcomes and related issues in the United States including surgical site infections [10,11], urinary tract infections [12], clostridium difficile [13], and hospital acquired pressure ulcers [14]. Like their European counterparts, some of these have included efforts to address the relationship between health care outcomes and costs [15].

The context of health care reform in national politics has become increasingly important for evaluation and improvement of hospital outcomes. This context relates to the process of health care reform and the development of software to define and analyze these outcomes. Despite increasing interest in the subject, research concerning the costs of adverse health care outcomes and efforts to address them have been hampered by the lack of major provider incentives for quality improvement and the difficulties of data collection in this area. Recent developments suggest that situation is changing in the United States.

Evidence is growing that the health care reform movement in the United States that was stimulated by the Obama Administration will attempt to reduce provider reimbursement for adverse clinical outcomes. A major driver of this process has been increased public awareness of the high level of health care spending in the United States. Within the health care reform debate, the Obama Administration has repeatedly emphasized the lack of connection between high levels of health care expenditures and outcomes such as life expectancy and mortality compared with other nations [16-18].

These issues have been complicated by the impact of the economic recession of 2008 and 2009. The need for increased government spending by the U.S. federal government to stimulate economic recovery has been constrained by the continued increase in public expenditures for health care through the Medicare and Medicaid programs [19]. In his Inaugural Address, President Obama called for higher quality of care at reduced costs [20].

In this context, it has been suggested that inpatient hospital complications generate large expenses in Europe and the United States. These complications are diagnoses which developed after admission to inpatient hospital beds. These complications are defined as harmful events or negative outcomes occurring during inpatient hospitalization that result from the processes of care and treatment rather than the natural progression of diseases [11].

Inpatient hospital complications are important because they can result in substantial adverse outcomes for patients. Events such as infections and circulatory complications cause considerable discomfort for patients, sometimes with life threatening consequences. These events disrupt the patients' recovery as well as their return to residential environments and employment. Because complications can extend hospital stays and consume additional resources, they can also block access to hospital beds for other patients.

Inpatient complications are also costly. They result in large expenditures for additional nursing time, pharmaceuticals, and tests before discharge is possible. Although the frequency of complications is often low, the total additional expenditures can be large at the provider and system wide levels [16].

Fortunately, the rising interest in improving the quality of care as a means of reducing costs are being supported by the availability of new resources for the collection and analysis of health care outcomes data. These include new administrative data categories which address outcomes such as inpatient hospital complications and computer software such as the Potentially Preventable Complications system developed by the 3M™ Corporation which was created to analyze these indicators [21]. The Potentially Preventable Complications system was developed to evaluate inpatient hospital outcomes based on administrative data. The system has been validated in preliminary studies by the New York State Department of Health and the Massachusetts Hospital Association. The system is currently being evaluated in a series of demonstration projects in the United States.

This objective of this study was to describe the Potentially Preventable Complications software as a tool for evaluating hospital inpatient outcomes. The study showed the use of this software to analyze the connection between health care outcomes and expenses in United States administrative data at the state and local levels. The study also described the use of the software to plan and implement interventions to reduce hospital complications in one U.S. metropolitan area.

The data used in this study are openly available. Administrative databases include patient specific data but may, in some cases, limit the availability of unique patient identifiers.

## Methods

### Potentially Preventable Complications

Potentially Preventable Complications is a system for categorizing and evaluating inpatient hospital complications developed by the 3M™ Corporation. The following material describes the specific components of this system within the context of hospital outcomes improvement.

The PPC system provides information concerning a full range of complications, as well as individual categories for health care providers [21]. The complications included in the system were identified through a review of existing literature, the diagnosis codes used in the Complications Screening Protocol developed by Iezzoni et al, and the Patient Safety Indicators developed by the Agency for Healthcare Research and Quality [22,23].

The full list of PPCs includes 64 mutually exclusive categories that were identified from ICD-9-CM procedure codes for secondary diagnoses. These diagnoses are identified as clinical conditions that were not the principal causes of hospital admission. The 64 categories have been condensed to a summary list of 35 PPCs. This process was carried out in order to support clinical management of related diagnoses in hospitals. It was based on clinical management needs, rather than scientific evidence concerning these diagnoses. 3M™ Health Information Services is currently developing a revised PPC algorithm based on ICD-10 codes. That process is scheduled for completion in 2011. The summary list follows in Table 1.

**Table 1:** PPC Summary Categories

01	Stroke & Intracranial Hemorrhage
02	Extreme CNS Complications

03	Acute Pulmonary Edema and Respiratory Failure with Mechanical Ventilation

04	Pneumonia & Other Lung Infections

05	Aspiration Pneumonia

06	Pulmonary Embolism

07	Shock

08	Congestive Heart Failure

09	Acute Myocardial Infarct

10	Ventricular Fibrillation/Cardiac Arrest

11	Peripheral Vascular Complications Except Venous Thrombosis

12	Venous Thrombosis

13	Major Gastrointestinal Complications with Transfusion or Significant Bleeding

14	Major Liver Complications

15	Clostridium Difficile Colitis

16	Urinary Tract Infection

17	Renal Failure with Dialysis

18	Post-Hemorrhage & Other Acute Anemia with Transfusion

19	Decubitus Ulcer

20	Septicemia & Severe Infections

21	Post-Op Wound Infection & Deep Wound Disruption with Procedure

22	Reopening Surgical Site

23	Post-Op Hemorrhage & Hematoma with Hem Cntrl Proc or I&D Proc

24	Accidental Puncture/Laceration during Invasive Procedure

25	Post-Procedure Foreign Bodies

26	Encephalopathy

27	Iatrogenic Pneumothrax

28	Mechanical Complication of Device, Implant & Graft

29	Inflammation & Other Complications of Devices, Implants or Grafts Except Vascular Infection

30	Infections Due to Central Venous Catheters

31	Obstetrical Hemorrhage with Transfusion

32	Obstetric Laceration & Other Trauma without Instrumentation

33	Obstetric Laceration & Other Trauma with Instrumentation

34	Major Puerperal Infection and Other Major Obstetric Complications

35	Post-Op Respiratory Failure with Tracheostomy

Under the Potentially Preventable Complication system, a number of diagnoses are excluded as non preventable. These include complications directly related to malignant diseases, multiple trauma, organ transplants, specific burns, and HIV related disorders. Because of their unique characteristics, neonates are also excluded. A number of these diagnoses are excluded by the algorithm as global exclusions, whether they appear as principal or secondary diagnoses. Others are excluded only when accompanied by another specific diagnosis [21].

The Potentially Preventable Complications system employs administrative hospital data. Within these data, the Present on Admission indicator is used to identify diagnoses which develop after admission to acute hospitals. The Present on Admission Indicator is applied to each secondary diagnosis of each hospital inpatient. Each of these diagnoses are identified as present on admission, not present on admission, or undetermined.

Use of the Present on Admission indicator on a widespread basis in the United States was made possible by National Uniform Billing Committee changes (UB 04) approved May 23, 2007. Under these changes, the standard inpatient claim form was modified to allow the submission of a Present on Admission indicator for each secondary diagnosis [24]. A number of state data bases have followed Medicare by requiring hospitals to use this indicator.

The validity of the Present on Admission indicator is based on the degree of compliance of hospital staffs with Medicare criteria for this indicator. It was mandated for use throughout the United States by Medicare in 2007. In New York State, this indicator has been used since the mid 1990s. It is assumed that the extensive experience of New York State and the Syracuse hospitals with the Present on Admission indicator contributed to the validity of related data used to define PPCs [25]. At the same time, this experience is based on unpublished data which has not benefited from extensive evaluation and scrutiny. For this reason, additional evaluation of the reliability of the Present on Admission data is necessary.

Under the Potentially Preventable Complications system, candidate diagnoses are those that are not excluded at the global or specific levels. These diagnoses are identified as not present on hospital admission. The candidate diagnoses are evaluated with respect to risk of complications based on reasons for admission and severity of illness. Evaluation of risk of complications based on reason for admission is related to hospital services provided to each patient. A major factor in this evaluation is the type of hospital service, whether medical or surgical, that a patient receives. The susceptibility of complications varies widely among medical and surgical patients. For example, among medicine patients, aspiration pneumonia is more likely to develop as a complication for a patient with stroke as a principal diagnosis than for one with urinary retention. The type of surgery is also a major influence on the likelihood of specific complications [21].

Risk of complications is also closely related to the severity of illness of each patient. Those hospitalized with severe secondary diagnoses or with multiple comorbidities have a higher risk of developing complications than those who do not [26,21]. The Potentially Preventable Complication system employs the All Patients Refined algorithm to determine severity of illness. This algorithm employs degree of illness based on principal and secondary diagnoses identified by (ICD-9-CM) medical record codes, patient age, and other factors [27].

The use of severity of illness to evaluate risk of hospital complications makes it possible to compare risk levels within or between hospitals under the Potentially Preventable Complications System. Within this context, comparisons can be developed within the same hospital, for different hospitals, or between hospitals and regional benchmark populations. For this reason, comparisons of populations need to be stratified by Diagnosis Related Group and severity of illness. This capability is limited by the fact that secondary diagnoses and comorbidities that are complications also influence severity of illness within Diagnosis Related Groups. For example, the development of pneumonia as a post admission complication could cause the severity of illness of a medical or surgical inpatient to increase from 1 (Minor) to 2 (Moderate). A post admission complication for urinary tract infection would have a similar impact. The impact of this phenomenon can be limited by the fact that inpatient complication rates are generally relatively low.

The Potentially Preventable Complications system has made it possible to evaluate the financial impact of these outcomes in acute hospitals. Because complications are identified by All Patients Refined Severity of Illness, it is possible to identify at risk populations and populations who experience each complication by DRG and severity. The hospital charges for these populations can then be compared using administrative data. These data are based on reported charges by hospital. Because charges are all inclusive, they can contain actual costs as well as provider mark ups. In the study cited, they were assumed to be generally representative of actual hospital costs. It must be emphasized, however, that the quantitative relationship varies by hospital because of differences in cost accounting and provider mark ups [24].

The Potentially Preventable Complications system is currently being tested at a number of hospitals in the United States. State governments and private organizations will be using the system for public and provider reporting of outcomes data.

### Potentially Preventable Complications Applied

The application of the Potentially Preventable Complications system to hospital quality improvement is illustrated by the experiences of the acute care facilities of Syracuse, New York. The metropolitan area of Syracuse, New York includes a resident population of 446,065 [28] and four urban hospitals. The hospitals, which generate approximately 70,000 discharges annually, have historically worked to improve efficiency and outcomes through their cooperative planning organization, the Hospital Executive Council [29].

The Syracuse hospitals became interested in using the Potentially Preventable Complications system for different reasons. One of these was the commitment of the Obama Administration to reducing health care costs by improving the quality of care. The Administration has suggested that reduction of complications will be an important part of its health care reform program. The Obama Administration has indicated that it will be developing financial mechanisms for reducing complications as part of the implementation of its health care reform program during 2011 and 2012. In New York State, reduction of complications through the use of PPCs has been included in the Governor's proposed budget for 2010 - 2011.

Another major influence on the Syracuse hospitals was the potential for internal cost savings. Preliminary inpatient data developed by the Hospital Executive Council and the Syracuse hospitals suggested that the actual costs of treating patients with PPCs was substantially higher than the costs of treating patients with the same APR DRGs and severity levels without the complications. The data were based on actual hospital costs rather than charges. These costs were developed using cost accounting systems at the individual hospitals.

In this study, the Potentially Preventable Complications categories were employed as a component of the Method. This was based on the need to test the use of these categories in addressing inpatient complications. It was not within the scope of this study to test or adjust the content of the PPC categories.

The program for reduction of Potentially Preventable Complications in the Syracuse hospitals was developed jointly by hospital staff members identified by the Chief Executive Officers, the Hospital Executive Council staff, and representatives of 3M™ Health Information Services. It included the following components.

Data Development and Distribution

Educational Program

Clinical Record Reviews

Selection of Objectives by PPC Category

Identification of Drivers of Complications

Identification and Implementation of Interventions

At the beginning of the project, the 3M™ Potentially Preventable Complications Software was still under development and could not be transferred. As a result, the initial monitoring data were produced directly by 3M™ and distributed in Syracuse through the Hospital Executive Council. These data included summary tables containing PPC frequencies and rates per at risk discharges for the individual hospitals and severity adjusted benchmark populations. These data were produced for the 35 PPC summary categories. They also included patient specific summary data for the hospital PPC and at risk populations. Initial data provided were for the periods January - December 2007 and January - June 2008.

The project also involved presentation of an educational program to key hospital staff members. The program involved teleconferences concerning the Potentially Preventable Complications System by senior staff of 3M™ Health Information Services to hospital administrators, physicians, and quality assurance staff. These presentations described the development and structure of the PPC algorithm, as well as its potential uses to improve outcomes and reduce costs.

Following the educational presentations, each hospital used the Potentially Preventable Complications information which had been provided to conduct reviews comparing the information produced by the software with clinical data in patient charts. At three of the hospitals, the data produced by the software was found to be consistent with data in the charts. At the fourth hospital, it was determined that the existence of numerous false positives in the complications data would require a recoding process. This process was necessary to verify the presence of inpatient complications based on documentation in the patient records.

The next phase of the process was identification of Potentially Preventable Complications categories that were to be the objectives of the quality assurance process. Within each of these categories, the hospitals identified drivers of the complications and developed interventions to address the drivers. These activities were followed by the implementation of interventions between October 2008 and March 2009.

The interventions developed in the Syracuse hospitals focused on urinary tract infections, pneumonia, clostridium difficile colitis, decubitus ulcer, pulmonary embolism, and post hemorrhagic and other acute anemia with transfusion. Each hospital identified specific PPCs that would be the focus of its interventions. These selections were based on historical complication rates, hospital resources, and quality assurance objectives. The data in Table 2 identify numbers of hospital complications and complication rates compared with severity adjusted benchmarks for each PPC that was a focus on an intervention by the participating hospitals.

**Table 2:** Potentially Preventable Complications Programs Implemented between October - December 2008 Syracuse Hospitals January - June 2008

	Discharges with Major PPC Total Cases		Major PPC Rate/1,000		% Difference	Significance Level
	Actual	Expected	Actual	Expected		
Urinary Tract Infection						
Crouse Hospital	43	55.9	5.43	7.05	-23.03	
Community General Hospital	46	41.3	12.65	11.34	11.49	
Pneumonia						
St. Joseph's Hospital Health Center	145	112.0	10.09	7.79	29.46	p < 0.05
Clostridium Difficile Colitis						
Community General Hospital	12	4.9	2.99	1.22	144.20	
Dccubitus Ulcer						
Crouse Hospital	6	7.1	0.71	0.85	-15.69	
St. Joseph's Hospital Health Center	31	13.0	3.41	1.43	138.47	p < 0.05
Pulmonary Embolism						
Crouse Hospital	12	5.1	1.53	0.65	133.80	p < 0.05
Post-Hemorrhage & Another Acute Anemia w Transfusion						
St. Joseph's Hospital Health Center	55	16.0	7.36	2.14	243.75	p < 0.05

Sources: Hospital Executive Council; 3M Health Information Systems.

Interventions addressing urinary tract infections at Community-General and Crouse hospitals focused on development of protocols for urine sample collection and nursing procedures regarding catheter insertion, removal, and care. Educational programs were developed to implement these procedures at the two hospitals. At Crouse Hospital, stickers were developed for attachment to patient charts to remind nurses of the need for vigilance in this area. At Community-General, stamps were placed in patient charts to remind physicians to reorder or discontinue the catheters. At both hospitals, interventions also included daily monitoring of patients for sites of infection and laboratory work. The Crouse and Community-General programs were implemented in October 2008.

For pneumonia at St. Joseph's Hospital Health Center, a large majority of the complications were produced by surgery, especially cardiac and orthopedic surgery. It was determined that the principal drivers of this complication were ventilator use and failure to implement early ambulation of post surgery patients. In order to address this complication, the hospital developed interventions addressing ventilator associated pneumonia in October 2008. Additional protocols addressing community-acquired pneumonia, which included expedited ambulation of patients after surgery, were also implemented.

For clostridium difficile colitis (c. diff.) at Community-General Hospital, the principal driver was determined to be environmental because this infection can remain in the environment for long periods of time and cultured from almost any surface. Complications of influenza were also a potential driver, since they disrupt normal intestinal flora, leading to an overgrowth of c diff. Interventions were identified for this PPC including improved cleaning procedures in treatment and patient rooms, isolation of at risk patients, hand washing with soap rather than sanitizers, and increased cleaning times. It involved cleaning procedures in treatment and patient rooms using bleach sufficient to address the resistant nature of this organism. These procedures were developed and implemented by the nursing and housekeeping staffs of the hospital.

Two of the hospitals developed planning processes focusing on decubitus ulcers as complications. The planning process at both hospitals identified a number of drivers of decubitus ulcer development including the lack of skin assessments for patients at the time of admission including staging of these complications, the lack of complete care plans for ulcer assessment, the need for reassessment of changes in the complication, and the lack of pressure sensitive mattresses on some units.

Interventions developed to address these needs focused on complete wound care planning including assessment at the time of admission and reassessments during the stay. At Crouse Hospital, these procedures were to be implemented by a multidisciplinary team. Interventions also included installation of pressure sensitive mattresses for all medical-surgical beds. Because planning activities at both Crouse Hospital and St. Joseph's Hospital Health Center were completed between during the third quarter of 2008, the interventions were implemented soon after.

At Crouse Hospital, the planning process identified the drivers of the pulmonary embolism complication as the lack of procedures to identify patients at risk and the lack of a protocol and education plan for management of these patients. Interventions focused on development of these procedures and updating orthopedic pathways for fractured hip, total hip replacement, and joint replacement to include them. The development of a protocol to address the range of patient diagnoses identified with a PPC of pulmonary embolism in the 3M data required more time than had been anticipated. As a result, the protocol was not finalized and implemented until the second quarter of 2009.

The planning process for post hemorrhagic and acute anemia involved record review issues at two acute care facilities. Crouse Hospital developed an analysis of transfusion cases using patient charts and determined that a minority actually addressed PPC criteria. Procedures for coding this condition were revised to ensure that data generated reflected actual experience. St. Joseph's Hospital Health Center identified coding issues differentiating expected and acute blood loss anemia. In addition, the Hospital identified the lack of standards of care for transfusions as potential drivers of this complication. To address this driver, standards of care and transfusion triggers for physicians were revised. These procedures were implemented in the fourth quarter of 2008.

## Results

The initial results of the study involved the development of financial data concerning Potentially Preventable Complications. The financial analysis included administrative charge data from the states of California and Maryland analyzed by the 3M Health Information Services staff. It also included a brief, summary analysis of actual cost data from the Syracuse hospitals evaluated by the Hospital Executive Council. These two analyses, based on different data sets and indicators, were intended to provide estimates concerning the impact of inpatient complications on hospital costs.

Analysis of the charge data involved editing of information submitted by hospitals. Patient data that were incomplete, or based on a discharge status of transferred to another acute care facility or expired were excluded. It has been estimated that these data involved a maximum of 6 percent of the records from each state. The resulting information from the California Office of Statewide Health Planning and Development was based on fiscal year 2008 (July 2007 - June 2008) and included 235 hospitals. The resulting information from the Maryland Health Services and Cost Review Commission was based on fiscal year 2008 (October 2005 - September 2006) and included 43 hospitals [24].

The information from Maryland included uniform categories of financial data that made it possible to estimate costs accurately from charge data. The California charge data did not include this level of structure and were evaluated using a charge to cost ratio of 1.264. A regression model was applied to both data sets to estimate average costs per patient by Potentially Preventable Complications. Examples of incremental costs added to patient expenses for PPCs with the highest frequencies in both states are identified in Table 3[24].

**Table 3:** Estimated Potentially Preventable Complications Cost Based on Hospital Charge Data States of California and Maryland

	California Utilization		Maryland Utilization	
	Number of Discharges	Incremental PPC Cost ($)	Number of Discharges	Incremental PPC Cost ($)
Acute Pulmonary Edema and Respiratory Failure without Ventilation	5,712	7,109	4,863	5,983
Pneumonia & Other Lung Infections	10,781	16,901	4,615	13,176
Congestive Heart Failure	5,922	5,801	2,296	3,910
Clostridium Difficile Colitis	2,478	25,401	1,282	16,709
Urinary Tract Infection	12,677	9,637	6,904	6,345
Cellulitis	2,907	4,950	1,435	2,346
Post-Op Hemorrhage & Hematoma w/o Hemorrhage Control Procedure or I&D Procedure	6,925	6,758	3,391	6,190

Source: Fuller, R.L., McCullough, E.C., Bao. M.Z., and Averill, R.F., 3M Health Information Systems, calculations using FY2008 Inpatient Acute Hospital Data, Maryland Health Services and Cost Review Commission.

The data in Table 3 indicate that the mean incremental costs per Potentially Preventable Complication were similar. It was notable that different data bases and need for separate statistical methods produced estimated incremental costs that did not vary greatly. At the patient level, the estimated impact of these costs was substantial. The incremental costs of the complications for these categories ranged from $4,950 to $25,401 in California and from $3,910 to $16,709 in Maryland [24]. These data included results from the study, rather than a separate analysis.

In the development of their demonstration program concerning Potentially Preventable Complications, the Hospital Executive Council and the Syracuse hospitals also produced estimates of the impact of these outcomes on hospital costs. Like the evaluations involving the California and Maryland data, these comparisons were based on inpatient populations with the same APR DRGs and severity of illness levels that experienced the PPCs and patients with the same risk characteristics who did not experience the PPCs. The Syracuse comparisons, however, were based on actual cost data from the hospitals. Through this stratification, to the extent possible, the differences in costs identified resulted from the complications involved, rather than variations in diagnoses and severity of illness of the populations.

Examples of the cost analyses from three of the Syracuse hospitals are summarized in Table 4. They include discharges, total costs, and mean costs per discharge for selected PPCs during the period before implementation of the demonstration program. The comparisons were based on patients with and without each PPC who were assigned to the same All Patients Refined Diagnosis Related Groups and severity of illness levels within these APR DRGs.

**Table 4:** Hospital Inpatient Cost Comparisons Patients With and Without Selected Complications Syracuse Hospitals January - June 2008

	Patients with PPC			Patients at Risk for PPC			Significance Level
	Number of Discharges	Total Cost	Mean Cost Per Discharge	Number of Discharges	Total Cost	Mean Cost Per Discharge	
Community General Hospital							
Urinary Tract Infection	40	829,599	20,740	842	9,806,054	11,646	p < 0.05
Clostridium Difficile Colitis	10	267,747	26,775	253	2,716,270	10,736	p < 0.05
Decubitus Ulcer	9	198,671	22,075	413	4,829,114	11,693	
Post Hemorrhage & Other Acute Anemia w Transfusion	13	201,658	15,512	373	5,723,682	15,345	p < 0.05
							
Crouse Hospital							
Urinary Tract Infection	35	844,579	24,131	3,601	14,906,383	4,140	p < 0.05
Decubitus Ulcer	5	165,222	33,044	421	2,250,905	5,347	
Pulmonary Embolism	9	164,630	18,292	266	2,148,818	8,078	
							
St. Joseph's Hospital Health Center							
Pneumonia	84	5,425,856	64,594	3,152	41,081,238	13,033	p < 0.05
Decubitus Ulcer	26	2,398,773	92,261	8,564	22,658,533	2,646	p < 0.05

Sources: Hospital Executive Council; 3M Health Information Systems.

The comparisons of actual costs from the Syracuse hospitals, like those from California and Maryland, suggested the substantial impact of complications on expenses for hospital inpatients. For patients with urinary tract infection, clostridium difficile colitis, and decubitus ulcer at Community-General Hospital, mean costs for patients with the complication were more than double those for the same at risk populations without it. For patients with urinary tract infection at Crouse Hospital, pneumonia at St. Joseph's Hospital Health Center, and decubitus ulcer as a PPC at both hospitals, expenses were several times those for at risk groups without the complications. A portion of this may have resulted from surgery patients and those with higher severity of illness at those hospitals. These comparisons were developed based on actual hospital costs, stratified to include patients with and without the PPC that were assigned to the same All Patients Refined Diagnosis Related Groups and severity of illness

A review of drivers of these costs at St. Joseph's Hospital Health Center and Crouse Hospital suggested that increased expenses for nursing, pharmaceuticals, and diagnostic testing were most responsible. These expenses were based on the cost of these resources related to lengths of stay at the hospital. Substantially longer stays for patients with the PPCs accounted for higher use of resources by this group. The analysis did not include separate analysis of the drugs consumed or the procedures associated with the adverse events.

Evaluation of the impact of Potentially Preventable Complications involved the use of severity adjusted benchmark data. Initial use of this information was based on California inpatient data for 2005 and 2006. Severity adjusted benchmarks from this source for medical and surgical inpatients at Crouse Hospital and St. Joseph's Hospital Health Center in Syracuse during 2008 are summarized in Table 5.

**Table 5:** Benchmark Rates of Major Potentially Preventable Complications Medical/Surgical PPCs January - September 2008 Annualized

		Range of Major PPC Rates/1,000 Discharges Based on Syracuse Hospitals Experience		
01	Stroke & Intracranial Hemorrhage	1.18	-	2.56

02	Extreme CNS Complications	0.44	-	0.85

03	Acute Pulmonary Edema and Respiratory Failure with Mechanical Ventilation	2.80	-	5.83

04	Pneumonia & Other Lung Infections	5.22	-	8.73

05	Aspiration Pneumonia	2.25	-	3.02

06	Pulmonary Embolism	0.65	-	0.88

07	Shock	1.74	-	3.26

08	Congestive Heart Failure	3.85	-	6.56

09	Acute Myocardial Infarct	2.28	-	3.73

10	Ventricular Fibrillation/Cardiac Arrest	2.89	-	5.27

11	Peripheral Vascular Complications Except Venous Thrombosis	0.37	-	0.94

12	Venous Thrombosis	1.62	-	2.77

13	Major Gastrointestinal Complications with Transfusion or Significant Bleeding	0.36	-	0.53

14	Major Liver Complications	0.43	-	0.73

15	Clostridium Difficile Colitis	1.13	-	1.66

16	Urinary Tract Infection	7.04	-	10.20

17	Renal Failure with Dialysis	0.40	-	1.11

18	Post-Hemorrhage & Other Acute Anemia with Transfusion	1.53	-	2.09

19	Decubitus Ulcer	0.84	-	1.34

20	Septicemia & Severe Infections	4.09	-	6.46

21	Post-Op Wound Infection & Deep Wound Disruption with Procedure	0.36	-	0.34

22	Reopening Surgical Site	0.97	-	1.71

23	Post-Op Hemorrhage & Hematoma with Hem Cntrl Proc or I&D Proc	0.78	-	2.32

24	Accidental Puncture/Laceration during Invasive Procedure	7.72	-	7.71

25	Post-Procedure Foreign Bodies	0.13	-	0.17

26	Encephalopathy	1.03	-	1.84

27	Iatrogenic Pneumothrax	0.47	-	0.70

28	Mechanical Complication of Device, Implant & Graft	0.62	-	1.11

29	Inflammation & Other Complications of Devices, Implants or Grafts Except Vascular Infection	1.74	-	3.12

30	Infections Due to Central Venous Catheters	0.00	-	0.00

Syracuse hospitals experience based on Crouse Hospital and St. Joseph's Hospital Health Center data applied to California statewide data, 2005 - 2006.

In addition to containing hospital benchmark data, this information comprised a summary of the incidence of major Potentially Preventable Complications in California, the largest inpatient state database in the United States. The data indicate that the highest complication rates per 1,000 hospital discharges were 7.04 - 10.20 for Urinary Tract Infections, 7.71 - 7.72 for Accidental Punctures, and 5.22 - 8.73 for Pneumonia and Other Lung Infections.

Data related to Potentially Preventable Complications in the Syracuse hospitals also involved early evaluation of programs for managing these outcomes that were implemented between October 2008 and March 2009. These comparisons involved data for January-June 2008, the period immediately prior to implementation of the projects, and January-March and April-June 2009, the most recent period for which coded inpatient data were available for the hospitals. This information is summarized in Table 6.

**Table 6:** Potentially Preventable Complications Programs Implemented Between October - December 2008 Syracuse Hospitals January - June 2008, January - June 2009

	Dates	Discharges with Major PPC Total Cases		Major PPC Rate/1,000 Discharges		% Difference	Significance Level
		**Actual**	**Expected**	**Actual**	**Expected**		

Urinary Tract Infection							

Crouse Hospital	Jan - Jun 08	43	55.9	5.43	7.05	-23.03	

	Jan - Mar 09	39	27.9	10.44	7.46	39.92	p < 0.05

	Apr - Jun 09	27	20.4	9.96	7.52	32.43	

							

Community General	Jan - Jun 08	46	41.3	12.65	11.34	11.49	

Hospital	Jan - Mar 09	13	18.7	7.56	10.91	-30.67	

	Apr - Jun 09	17	20.0	10.02	11.79	-15.06	

							

Pneumonia							

St. Joseph's Hospital	Jan - Jun 08	145	112.0	10.09	7.79	29.46	p < 0.05

Health Center	Jan - Mar 09	42	31.0	12.34	9.11	35.48	

	Apr - Jun 09	34	27.0	11.69	9.28	25.93	

							

Clostridium Difficile Colitis							

Community General	Jan - Jun 08	12	4.9	2.99	1.22	144.20	

Hospital	Jan - Mar 09	7	2.3	3.73	1.21	209.10	

	Apr - Jun 09	4	2.5	2.13	1.32	60.99	

							

Decubitus Ulcer							

Crouse Hospital	Jan - Jun 08	6	7.1	0.71	0.85	-15.69	

	Jan - Mar 09	5	3.1	1.28	0.79	62.57	

	Apr - Jun 09	3	2.3	1.06	0.83	28.58	

							

St. Joseph's Hospital	Jan - Jun 08	31	13.0	3.41	1.43	138.47	p < 0.05

Health Center	Jan - Mar 09	9	6.0	2.12	1.41	50.00	

	Apr - Jun 09	7	5.0	1.95	1.39	39.99	

							

Pulmonary Embolism							

Crouse Hospital	Jan - Jun 08	12	5.1	1.53	0.65	133.80	p < 0.05

	Jan - Mar 09	5	2.5	1.32	0.65	101.44	

	Apr - Jun 09	2	1.8	0.73	0.67	9.24	

							

Post-Hemorrhage & Other Acute Anemia w Transfusion							

St. Joseph's Hospital	Jan - Jun 08	55	16.0	7.36	2.14	243.75	p < 0.05

Health Center	Jan - Mar 09	11	8.0	3.04	2.21	37.50	

	Apr - Jun 09	5	7.0	1.67	2.34	-28.57	

Sources: Hospital Executive Council; 3M Health Information Systems.

This information demonstrated that, within these time frames, complication rates had declined slightly for three of the initiatives. These included decubitus ulcer, and post hemorrhage/acute anemia at St. Joseph's Hospital Health Center and pulmonary embolism at Crouse Hospital. For these projects, differences between hospital and severity adjusted benchmark rates fell from significant to nonsignificant levels. The rate for urinary tract infection at Community-General Hospital declined from nonsignificantly higher to nonsignificantly lower than the benchmark. These declines resulted from before and after comparisons, rather than the use of control groups. In the participating hospitals, it was not possible to develop control groups within the scope of the study.

These comparisons also demonstrated that differences between hospital and severity adjusted PPC rates increased for urinary tract infection and decubitus ulcer at Crouse Hospital and for clostridium difficile colitis at Community-General Hospital. Only the increase for urinary tract infection reached a significant level. These declines resulted from before and after comparisons, rather than the use of control groups.

These data suggested that, based on the average cost differences identified in Table 4, the financial impact of managing complications could be considerable. This point was based on simple calculations of the impact of changes in complications at the Syracuse hospitals during the period of the study, rather than a specific analysis. For example, declines in PPC rates at St. Joseph's Hospital Health Center between January-June 2008 and January-March 2009 reduced the first quarter costs for patients at risk of pneumonia by $386,707.50 and for those at risk of decubitus ulcer by $134,422.5. These expenses would translate into annualized savings of $1,546,830 for pneumonia and $537,690 for decubitus ulcer. For Crouse Hospital, the decline in complication rates produced a first quarter savings of $20,218. For Community-General Hospital, the decline in complications for urinary tract infections produced a savings $90,940.

The demonstration project in Syracuse will generate additional information concerning the impact of clinical interventions on a range of inpatient complications within urban, general hospitals. It is projected to conclude at the end of 2010.

## Discussion

This study described, in a preliminary manner, the use of the Potentially Preventable Complications system to evaluate hospital complications, related costs, and interventions to address these outcomes. It approached the subject in a preliminary manner because this relationship is only beginning to be explored. The study included simple cost estimates of the impact of complications on hospital resource use based on administrative data. These estimates were based on statewide charge data and hospital actual costs concerning direct expenses for specific hospitals. The study also described use of the system to plan and implement interventions to reduce complications.

The early Dutch research on this subject has been interesting. It suggests that there are important cost implications of a wide range of adverse health care outcomes. This type of wide view of the subject is extremely necessary given current limitations on health care resources. More studies of this nature will be of great benefit to health care policy makers and providers.

The research from the United States presented in the article was of a more focused nature. It involved a single category of adverse outcomes, complications that develop within hospitals after inpatient admission. As an example, the complications data suggested what will be necessary if the management of health care outcomes is to move from the shadows where it has resided to become a major focus for cost containment, as well as the improvement of care.

In the United States, hospital reimbursement is based on prospective payments per discharge for public and private payors. Under this system, hospitals are paid a relatively fixed rate per patient. This encourages efficiency through reduction of complications, extended stays, and other developments which consume hospital resources. The current system includes a small penalty for the presence of inpatient complications. The amount of this penalty is much lower than the potential excess costs related to complications that were identified in this study.

In this context, the developing experience with inpatient hospital complications in the United States demonstrates the resources that are needed for this to occur. At the technical level, these include categories within administrative data, such as the Present on Admission indicator, that make it possible to collect and analyze outcomes data at the provider and regional levels. These resources also include software that focuses on the indicator through a sophisticated system of logic. It seems clear that the Potentially Preventable Complications algorithm, although still under development, contains the exclusions and criteria necessary to convert administrative data, including the Present on Admission indicator, to usable information. The linkage between the PPC software and the All Patients Refined Severity of Illness System produces the ability to analyze and compare outcomes data by level of illness within hospitals and large populations. The availability of this resource is helping to move quality assurance staff in hospitals like those in Syracuse from record keepers to clinical managers.

Current information also suggests that efforts to improve health care outcomes such as complications in the United States will benefit from the historical pressure to reduce health care costs which have been compounded by the impact of the recent economic recession. It seems clear from recent pronouncements from Washington that the Obama Administration believes strongly that there is a connection between health care outcomes and costs. Complications are not the only indicator that is benefiting from the attention. Reduction of hospital readmissions has also become a focus of attention. The federal Medicare program is interested in redesigning hospital reimbursement to address this objective [30].

## Conclusions

All of this suggests that, after years of struggling for greater attention within the provision of health care and the policy making that surrounds it, the monitoring and management of quality and outcomes may be about to move to the forefront. The development of the Potentially Preventable Complications algorithm is an example of this process. Historically, Europe and the United States have been the major sources of research and innovation concerning health care utilization and outcomes. The new opportunities can benefit both sides of the Atlantic if commitments are made to ensure that the necessary data and analytic tools are available. Now is the time to ensure that preliminary efforts, such as those described in this article, receive adequate support.

## Competing interests

The authors declare that they have no competing interests.

## Authors' contributions

RJL participated in planning the analysis, developed the data concerning the costs of complications, and coordinated the implementation of interventions in the hospitals of Syracuse, New York. GPW developed the structure of the study, contributed to the collection of the literature, and participated in planning the analysis. Both RJL and GPW developed the revisions to the manuscript which resulted in the final version. Each of the authors read and approved the final manuscript.

## Authors' information

Dr. Ronald J. Lagoe (1)

Prof. dr. Gert P. Westert (2,3)

1 Hospital Executive Council, PO Box 35089, University Station, Syracuse, New York, 13235, USA

2 Tilburg University, TRANZO, PO Box 90153, 5000 LE Tilburg, The Netherlands

3 RIVM National Institute for Public Health and the Environment, PO Box 1, 3720 BA Bilthoven, The Netherlands

## Pre-publication history

The pre-publication history for this paper can be accessed here:

http://www.biomedcentral.com/1472-6963/10/200/prepub

## References

[B1] Williams SC Quality of care in U.S. hospitals reflected by standardized measures 2002 - 2004 New England Journal of Medicine 2005 353 3 255 264 10.1056/NEJMsa043778 16034011

[B2] Hoonhout LH deBruijne MC Wagner C Zegers M Waaijman R Spreeuwenberg P Asscheman H van der Wal G Tulder van MW Direct medical costs of adverse events in Dutch hospitals BMC Health Services Research 2009 9 27 10.1186/1472-6963-9-27 19203365 PMC2645386

[B3] Zegers M deBruijne MC Wagner C Hoonhout LHF Waaijman R Smits M Adverse events and potentially preventable deaths in Dutch hospitals: results of a retrospective review study Quality and Safety in Health Care 2009 18 4 297 302 10.1136/qshc.2007.025924 19651935

[B4] Gianino MM Vallino A Minniti D Abbona F Mineccia C Silvaplana P Zotti CM A model for calculating costs of hospital-acquired infections: an Italian experience Journal of Health Organization Management 2007 21 1 39 53 10.1108/14777260710732259 17455811

[B5] Venditti M Falcone M Corrao S Licata G Serra P Outcomes of patients hospitalized with community - acquired, health care - associated, and hospital - acquired pneumonia Annals of Internal Medicine 2009 150 1 19 26 19124816 10.7326/0003-4819-150-1-200901060-00005

[B6] Wolkewitz M Vonberg RP Grundmann H Beyersmann J Gastmeir P Barwolff S Geffers C Behnke M Ruden H Schumacher M Risk factors for the development of nosocomial pneumonia and mortality on intensive care units: application of competing risks models Critical Care 2008 12 2 134 10.1186/cc6852 18384672 PMC2447589

[B7] Sheng WH Wang JT Lu DC Chie WC Chen YC Chang SC Comparative impact of hospital - acquired infections on medical costs, length of hospital stay, and outcome between community hospitals and medical centres Journal of Hospital Infections 2005 59 3 205 214 10.1016/j.jhin.2004.06.003 15694977

[B8] Gunningberg L Stotts NA Tracking quality over time: what do pressure ulcer data show? International Journal for Quality in Health Care 2008 20 4 246 253 10.1093/intqhc/mzn009 18390902

[B9] Vincent C Neale G Woloshynowych M Adverse events in British hospitals; preliminary retrospective record review British Medical Journal 2001 322 517 519 10.1136/bmj.322.7285.517 11230064 PMC26554

[B10] Kirkland KB Briggs JP Trivette SL Wilkinson WE Sexton DJ The impact of surgical site infections in the 1990s: attributable mortality, excess length of hospitalization, and extra costs Infection Control and Hospital Epidemiology 199 20 725 730 10.1086/501572 10580621

[B11] Peng M Kurtz MS Johannes RS Adverse outcomes from Hospital-acquired infection in Pennsylvania cannot be attributed to increased risk on admission American Journal of Medical Quality 2006 21 6 Supplement 17S 28S 10.1177/1062860606294632 17077415

[B12] Foxman B Epidemiology of urinary tract infections: incidence, morbidity, and economic costs American Journal of Medicine 2002 113 Supplement 1A 5S 13S 10.1016/S0002-9343(02)01054-9 12113866

[B13] Zerey M Paton BL Lincourt AE Gersin KS Kercher KW Heniford BT The burden of clostridium difficile in surgical patients in the United States Surgical Infection 2007 8 6 553 556 10.1089/sur.2007.9978 18171114

[B14] Chicano SG Drolshagen C Reducing hospital - acquired pressure ulcers Journal of Wound, Ostomy, and Continence Nursing 2009 36 1 45 50 10.1097/01.WON.0000345175.51117.ca 19155823

[B15] Aaron HJ Waste: We know you are out there New England Journal of Medicine 2008 359 18 1865 1867 10.1056/NEJMp0807204 18971487

[B16] Aaron HJ Ginsburg PB Is health care spending excessive? If so, what can we do about it? Health Affairs 2009 28 5 1260 1275 10.1377/hlthaff.28.5.1260 19738241

[B17] Skinner J Chandra A Goodman D Fisher ES The elusive connection between health care spending and quality Health Affairs 2009 28 1 w119 w123 10.1377/hlthaff.28.1.w119 19056756 PMC2811530

[B18] Farrell D Kocher B Laboissiere P Parker S Accounting for the cost of U.S. health care: A new look at why Americans spend more 2008 Washington, D.C.: McKinsey Global Institute

[B19] Marcus A Bending the curve: the twists and turns Health Affairs 2009 28 5 1256 1258 10.1377/hlthaff.28.5.1256 19738239

[B20] Obama B Presidential inaugural address, 2009 2009 Washington, D.C.: The White House

[B21] Hughes JS Averill RF Goldfield NJ Identifying potentially preventable complications using a present on admission indicator Health Care Financing Review 2006 27 3 63 82 17290649 PMC4194950

[B22] Iezzoni LI Daley J Heeren T Foley SM Fisher ES Duncan C Hughes JS Coffman GA Identifying complications of care using administrative data Medical Care 1994 32 7 700 715 10.1097/00005650-199407000-00004 8028405

[B23] Agency for Health Care Research and Quality National healthcare quality report 2003 U.S. Department of Health and Human Services Rockville, MD

[B24] Fuller RL McCullough EC Bao MZ Averill RF Estimating the costs of potentially preventable hospital acquired complications Health Care Financing Review 2009 30 4 17 32 19719030 PMC4195062

[B25] Massachusetts Hospital Association Position concerning potentially preventable complications 2010

[B26] Daley J Henderson WG Khuri SF Risk - adjusted surgical outcomes Annual review of medicine 2001 52 275 287 10.1146/annurev.med.52.1.275 11160779

[B27] Averill RF Goldfield NI Muldoon J A closer look at all patients refined DRGs J AHIMA 2002 10 46 50 12469662

[B28] New York Statistical Information System Population projections for New York State counties 2008 Ithaca, New York: Cornell University

[B29] Lagoe R Pasinski T Kronenberg P Quinn T Schaengold P Linking health services at the community level Canada Health Care Quarterly 2006 9 3 60 65 10.12927/hcq..18229 16826768

[B30] Averill RF McCullough EC Hughes JS Goldfield NI Vertrees JC Fuller RL Redesigning the Medicare inpatient PPS to reduce payments to hospitals with high readmission rates Health Care Financing Review 2009 30 4 1 15 19719029 PMC4195060

